# Use of Zoo Mice in Study of Lymphocytic Choriomeningitis Mammarenavirus, Germany 

**DOI:** 10.3201/eid3001.230334

**Published:** 2024-01

**Authors:** Joë̈lle Goü̈y de Bellocq, Stuart J.E. Baird, Alena Fornůsková

**Affiliations:** Institute of Vertebrate Biology of the Czech Academy of Sciences, Brno, Czech Republic

**Keywords:** lymphocytic choriomeningitis mammarenavirus, LCMV, viruses, house mouse, zoo

**To the Editor:** Mehl et al. (*1*) report high prevalence of lymphocytic choriomeningitis mammarenavirus (LCMV) in mice captured in a zoo in Germany; mice were screened after detection of LCMV in a golden lion tamarin. Similarly high LCMV prevalences have been detected in mouse breeding facilities (MBFs) (*2*). Mehl et al. suggested the zoo LCMV strains do not support the biogeographic hypothesis for LCMV distribution proposed by Fornůsková et al. (*3*). We feel obliged to point out that data collected from zoos cannot inform regarding biogeographic hypotheses, either way.

Fornůsková et al. (*3*) surveyed LCMV in natural (low-prevalence) house mouse populations. Their findings showed that an apparently random distribution of LCMV lineages in human infections, taken from public databases, is resolved by tracing viral origins not to diagnosing institutes, but instead through patient history. With origin tracing, most current data are consistent with the hypothesis that LCMV lineage I (sensu; *4*) originates in the range of *Mus musculus domesticus* mice, whereas LCMV lineage II originates in the range of *M. m. musculus* mice.

Regarding the infected lion tamarin (*1*), numerous LCMV infections have been reported in zoo primates (*5*); zoos in Europe exchange primates, including lion tamarins. Regarding the zoo-captured mice, zoos either maintain their own MBFs or receive live mice from external MBFs to feed reptiles, raptors, and other small carnivores. Presence of MBF mice in zoos breaks origin tracing of wild mouse pathogens because domesticated mice are crosses of 3 wild subspecies; origins of strains used to mass-produce animal food are unregulated. Mehl et al. (*1*) found multiple LCMV strains in a high-density host-pathogen transport hub. Whether such hubs might in the future lead to a breakdown in the current biogeographic pattern of LCMV lineages remains an open question.
